# Editorial: Molecular and Cellular Mechanisms in Reproduction and Early Development

**DOI:** 10.3389/fcell.2019.00036

**Published:** 2019-03-20

**Authors:** Rafael A. Fissore, Adam Burton, Karin Lykke-Hartmann

**Affiliations:** ^1^Department of Veterinary and Animal Sciences, University of Massachusetts, Amherst, MA, United States; ^2^Institute of Epigenetics and Stem Cells, Helmholtz Zentrum München, München, Germany; ^3^Department of Biomedicine, C Aarhus University, Aarhus, Denmark; ^4^Department of Clinical Medicine, Aarhus University, Aarhus, Denmark; ^5^Department of Clinical Genetics, Aarhus University Hospital, Aarhus, Denmark

**Keywords:** male and female gametes, Ca^2+^ signaling, fertilization, chromosomes, histones, folate, human pluripotent stem cells

The growth and division of gamete cells are prerequisites for successful fertility and have received increasing attention in research communities (Skinner, 2005; Da Silva-Buttkus et al., 2008; Shah et al., 2018).

In the early 1950s, Austin and Chang independently described the changes that are required for the sperm to fertilize oocytes *in vivo*. During capacitation, sperm undergo a change in the motility pattern called hyperactivation (Yanagimachi, 1970). In this issue of Frontiers, a comprehensive review addresses the importance of this in the male gamete and the changes that occur in sperm during their transit through the male and female reproductive tracts by complex signaling cascades with focus on the principal molecular mechanisms that govern human sperm capacitation (Puga Molina et al). Sperm are both transcriptionally and translationally silent, therefore post-translational modifications are essential to regulate their function. In this issue for Frontiers, a study shows that that O-GlcNAc transferase (OGT), the enzyme responsible for O-GlcNAcylation, is present in the testis, epididymis, and immature caput sperm, which indicates that modulation of O-GlcNAcylation takes place during sperm maturation and suggest a role for this post-translational modification in this process (Tourzani et al).

Already during embryo development in the mother's uterus, the pool of oocytes is established, maintained, and stored. The pool of the earliest primordial (resting) follicles is almost completely laid down in ovaries during fetal life and constitutes at any moment in time the reproductive potential of a female (Skinner, 2005; Da Silva-Buttkus et al., 2008; Shah et al., 2018). Once activated, the primordial follicles grow in size, and the flat layer of surrounding granulosa cells, which is characteristic for primordial follicles, transforms into cubic granulosa cells, typical of activated primary follicles (Skinner, 2005; Da Silva-Buttkus et al., 2008; Shah et al., 2018). Activation of the primordial follicles occurs in a hormone-independent manner (Edson et al., 2009; Tingen et al., 2009). The PI3K/Akt/mTOR pathways are known players in this transition (Goto et al., 2007; Reddy et al., 2008; Jagarlamudi et al., 2009; Adhikari et al., 2013; Makker et al., 2014; Cheng et al., 2015b; Hsueh et al., 2015), and are regulated by phosphatase and tensin homologs deleted on chromosome 10 (PTEN), the tuberous sclerosis complex (TSC1/2) and recently, the HIPPO signaling (Kawamura et al., 2013; Cheng et al., 2015a; Kawashima and Kawamura, 2018), synergistically with the phosphatidylinositol 3-kinase (PI3K)/AKT pathway (Grosbois and Demeestere, 2018). Several contributions in this issue for Frontiers describe new signaling pathwaysas potential regulators of the primordial-to-primary transition in human follicles, with a new view on how androgens might contribute (Ernst et al.; Steffensen et al.).

Egg activation at fertilization in mammalian eggs is caused by a series of transient increases in the cytosolic free calcium (Ca^2+^) concentration, referred to as Ca^2+^ oscillations (Stricker, 1999). These Ca^2+^ oscillations are initiated by a sperm specific phospholipase Czeta isoform, PLCζ that hydrolyses its substrate PIP_2_ to produce the Ca^2+^ releasing messenger InsP_3_. In this issue of Frontiers, a study shows that PLCζ induce Ca^2+^ Oscillations in mouse eggs, which involve a positive feedback cycle of Ca^2+^ induces InsP_3_ formation from cytoplasmic PIP_2_ (Sanders et al). This manuscript also suggests that the site of InsP3 production by PLCζ is from PIP_2_-containig cytoplasmic vesicles spread throughout the cytoplasm, which is diametrically different from the site of PIP_2_ hydrolysis by other PLCs. Oocyte maturation is associated with changes in the electrical properties of the plasma membrane and alterations in the function and distribution of ion channels. Therefore, variations on the pattern of expression, distribution, and function of ion channels and transporters during oocyte maturation are fundamental to reproductive success. In this issue for Frontiers, a review comprehensively discusses the role of ion channels during oocyte maturation, fertilization and early embryonic development, and how ion channel studies in *Xenopus* oocytes, an extensively studied model of oocyte maturation, translate into a greater understanding of the role of ion channels in mammalian oocyte physiology (Carvacho et al).

Chromosome dynamics during meiotic prophase I are associated with a series of major events such as chromosomal reorganization and condensation, pairing/synapsis and recombination of the homologs, and chromosome movements at the nuclear envelope (NE). The linker of nucleoskeleton and cytoskeleton (LINC) complexes are important constituents of the NE that facilitate in the transfer of cytoskeletal forces across the NE to individual chromosomes. In this issue for Frontiers, a review summarizes the findings of recent studies on meiosis-specific constituents and modifications of the NE and corresponding nucleoplasmic/cytoplasmic adaptors being involved in NE-associated movement of meiotic chromosomes, as well as describing the potential molecular network of transferring cytoplasm-derived forces into meiotic chromosomes in model organisms (Zeng et al.), aiming to increase our understanding of the NE-associated meiotic chromosomal movements in plants.

The newly formed 1-cell embryo (the zygote) undergoes its first mitotic cell division to form the 2-cell stage embryo, a transition mainly controlled by maternal factors stored in the oocyte (Zheng and Liu, 2012). Folates have been shown to play a crucial role for proper development of the embryo as folate deficiency has been associated with reduced developmental capacity such as increased risk of fetal neural tube defects and spontaneous abortion. In this issue for Frontiers, a study shows that maternally contributed FOLR1 protein appears to maintain ovarian functions, and contribute to preimplantation development combined with embryonically synthesized FOLR1 (Strandgaard et al).

Packaging DNA into chromatin allows for mitosis and meiosis, prevents chromosome breakage and controls gene expression and DNA replication (Borsos and Torres-Padilla, 2016). Histones contribute to eukaryotic chromatin structure and function in a well-known manner (Harr et al., 2016). Interestingly, free histones also have antimicrobial functions (Kawasaki and Iwamuro, 2008). For example, histones in amniotic fluid appear to fight bacteria by neutralizing the lipopolysaccharide (LPS) of microbes that gain access to this fluid (Witkin et al., 2011). The possible benefits of mitigating extracellular histone cytotoxicity have been outlined for the reproductive tract and other organs, however, in this issue of Frontiers, an opinion article reassesses previously published data to support the notion that uterine histone secretion fosters early embryo development in multiple ways (Van Winkle).

The regulation of signaling pathways by Ca^2+^ occurring at the earliest stages of development is not only important in fertilization, but also for human pluripotent stem cells (hPSC) maintenance (Todorova et al., 2009). The Ca^2+^ P-type ATPases, the plasma membrane calcium ATPases (PMCAs) and the sarco/endoplasmic reticulum Ca^2+^ ATPase (SERCAs), which reside in different compartments of the cell and along with other Ca^2+^ transporting system, contribute to the regulation of the intracellular Ca^2+^ concentration. In this issue for Frontiers, a study uses hPSCs to generated neural stem cells (NSCs) of the central and peripheral nervous system and investigated the main neural progenitor states for the presence of PMCAs using RNA sequencing (RNA-seq) and immunofluorescent labeling, and show that dynamic change in ATPase expression correlates directly with the stage of differentiation (Chen et al). These data have important implications for understanding the role of Ca^2+^ in development and potentially how disease states, which disrupt Ca^2+^ homeostasis, can result in global cellular dysfunction.

We hope that the articles in this topic will be of interest to researchers working in development and cell biology, providing basis for further discussion on this area to initiate new research questions that will contribute to our further understanding of cell growth and division in developmental contexts.

## Author Contributions

KL-H was the Guest editor of this Research Topic, inviting co-editors AB and RF working with them to define the subjects to be treated. They identified and invited leaders in specific research fields to contribute their work to the Research Topic. They acted as handling editors of manuscripts in the topic. KL-H wrote the Editorial with input from the other co-editors.

### Conflict of Interest Statement

The authors declare that the research was conducted in the absence of any commercial or financial relationships that could be construed as a potential conflict of interest.
